# FOXO3a promotes odontoblast differentiation in human dental pulp cells in coordination with RUNX2

**DOI:** 10.1590/1678-7765-2026-0114

**Published:** 2026-08-14

**Authors:** Su-Kyung Son, Jung-Sun Moon, Na-Ri Jung, Jee-Hae Kang, Bin-Na Lee, Sun-Hun Kim, Min-Seok Kim

**Affiliations:** 1 Chonnam National University School of Dentistry Dental Science Research Institute Gwangju Republic of Korea Chonnam National University, School of Dentistry, Dental Science Research Institute, Gwangju, Republic of Korea.

**Keywords:** FOXO3a, Odontoblast differentiation, RUNX2, DSPP, AKT signaling, Human dental pulp cells

## Abstract

**Introduction:**

Forkhead box O3a (FOXO3a) is a stress-responsive transcription factor involved in cellular differentiation across multiple tissues. However, its role in odontoblast differentiation and dentin matrix formation remains poorly defined.

**Objective:**

This study investigated the functional involvement of FOXO3a in odontoblast differentiation and its association with key odontogenic regulators.

**Methodology:**

Human dental pulp cells (HDPCs) were induced to undergo odontoblast differentiation *in vitro*. *FOXO3a* gene and protein expression patterns were examined during differentiation and in postnatal Sprague Dawley rat molars. Gain- and loss-of-function approaches assessed the role of *FOXO3a* in odontogenic differentiation. Odontoblast marker expression, alkaline phosphatase activity, and mineralized matrix formation were evaluated. RUNX2 transcriptional activity was analyzed using promoter reporter assays, and the interactions between FOXO3a and AKT signaling were examined through pharmacological modulation.

**Results:**

FOXO3a expression increased during early odontoblast differentiation. *FOXO3a* silencing reduced odontogenic marker expression, including *DSPP*, *DMP1*, and *RUNX2*, and significantly attenuated mineralization, whereas *FOXO3a* overexpression enhanced these effects. FOXO3a increased RUNX2 transcriptional activity and cooperated with RUNX2 to regulate *DSPP* promoter activity. AKT activation partially rescued odontogenic marker expression in FOXO3a-silenced cells, supporting functional interplay between these pathways.

**Conclusions:**

FOXO3a contributes to odontoblast differentiation by coordinating with RUNX2-associated transcriptional activity and interacting dynamically with AKT signaling. FOXO3a may represent a regulatory component of dentin matrix gene expression, relevant to regenerative endodontic applications.

## Introduction

Dental pulp tissue plays a central role in dentin regeneration through the differentiation of dental pulp cells into odontoblasts.^1^ This process is controlled by transcriptional networks responding to environmental cues.^2-4^ Although multiple signaling pathways regulating odontoblast differentiation have been identified, the transcriptional mechanisms integrating stress-responsive signaling with odontogenic differentiation and dentin matrix formation remain incompletely understood.

Forkhead box O (FOXO) transcription factors regulate diverse cellular functions, including proliferation, differentiation, and stress responses.^5,6^ Among FOXO family members, FOXO3a has been implicated in osteogenic differentiation and tissue homeostasis through interactions with key transcriptional regulators and signaling pathways.^7-9^ The functional role of FOXO3a appears to be highly context-dependent. Previous studies have reported that FOXO3a overexpression in MC3T3-E1 cells suppresses mineralization by downregulating calcium transport-related genes.^8^ In contrast, resveratrol has been shown to promote osteogenic differentiation of human mesenchymal stem cells through activation of the master osteogenic transcription factor runt-related transcription factor 2 (RUNX2) via SIRT1-dependent interaction with FOXO3a.^7^ In addition, nicotinamide has been reported to promote osteoblast differentiation through activation of the SIRT3–FOXO3a signaling pathway and enhancement of the mitochondrial antioxidant defense system.^9^ However, despite biological similarities between osteoblasts and odontoblasts, the role of FOXO3a in odontoblast differentiation and dentin matrix gene expression remains largely unexplored.

To date, only limited evidence has addressed the involvement of FOXO3a in dental pulp and odontoblast biology. Previous studies have shown that FOXO3a participates in inflammatory responses in human dental pulp cells (HDPCs), and another study suggested that FOXO3a regulates inflammation-induced autophagy in odontoblasts.^6,10^ However, whether FOXO3a is dynamically regulated during odontoblast differentiation, whether it functionally contributes to dentin matrix gene expression and mineralized matrix formation, and how it may be linked to odontogenic transcriptional regulators remain unclear.

RUNX2 is a key regulator of mineralized tissue formation and is required for early odontoblast differentiation.^11-13^ RUNX2 contributes to the transcriptional regulation of odontoblast-associated genes, including DSPP, but its activity is dynamically modulated by upstream signaling pathways, including protein kinase B (AKT)-related signaling.^14,15^ Because FOXO3a activity is also regulated by AKT-mediated phosphorylation, FOXO3a may contribute to odontogenic transcriptional programs by functionally coordinating with RUNX2-associated transcriptional activity within an AKT-responsive signaling context.^16^

In this study, the function of FOXO3a in odontoblast differentiation was investigated using HDPCs. In addition, postnatal rat molar tissues were analyzed to provide an *in vivo* developmental context for FOXO3a expression in the dentin–pulp complex. Specifically, the temporal regulation of FOXO3a during odontoblast differentiation was examined, and the effects of FOXO3a loss and gain of function on odontoblast marker expression and mineralized matrix formation were determined. The functional relationship of FOXO3a with RUNX2-associated transcriptional activity and AKT signaling was explored. Collectively, these analyses provide evidence that FOXO3a functions as a regulatory component of odontoblast differentiation and dentin matrix gene expression.

## Methodology

### Cell Isolation, Culture and Odontoblast Differentiation

HDPCs were isolated from healthy premolars extracted for orthodontic purposes after obtaining informed consent, following protocols approved by the Institutional Review Board of Chonnam National University Dental Hospital, Gwangju, Korea (IRB No. CNUDH-2016-009). Cells from three independent donors were used. HDPCs were cultured in α-minimum essential medium (α-MEM; Gibco, Thermo Fisher Scientific Inc., Waltham, Massachusetts, USA) supplemented with 10% fetal bovine serum (FBS; Gibco) and 1% penicillin–streptomycin (Gibco) at 37°C in a humidified atmosphere containing 5% CO_2_. HDPCs were characterized by flow cytometry in our previous study using the same experimental cell source.^17^ The characterization profiles confirming a mesenchymal stem cell-like surface marker pattern are provided in Supplementary Figure 2 of the study published by Shin, et al.^17^ (2025). Odontoblast differentiation was induced using α-MEM containing 10 mM β-glycerophosphate, 50 μg/mL ascorbic acid, and 100 nM dexamethasone (Sigma-Aldrich, St. Louis, MO, USA). The differentiation medium was changed every 3 days, and cells were harvested at the indicated time points.

### Gene silencing and overexpression

HDPCs were seeded in 6-well plates at 1–2 × 10⁵ cells per well. To silence FOXO3a expression, cells were transfected with FOXO3a-specific small interfering RNA (siRNA; 20 nM; Bioneer Corporation, Daejeon, Korea) or non-targeting control siRNA using Lipofectamine RNAiMAX^®^ reagent (Invitrogen, Carlsbad, CA, USA) according to the manufacturer’s instructions. At 24 h post-transfection, the medium was replaced with odontogenic differentiation medium, and cells were cultured for the indicated durations. For FOXO3a overexpression, HDPCs were transfected with 1 µg of pcDNA4-FOXO3a plasmid using Lipofectamine LTX reagent (Invitrogen) according to the manufacturer’s instructions. FOXO3a expression was driven by the CMV promoter, and the corresponding empty pcDNA4 vector was used as a control. Plasmid transfection efficiency was assessed in parallel using an EGFP-expressing control plasmid under the same transfection conditions. After 24 h, nuclei were stained with DAPI, and EGFP-positive and DAPI-positive cells were counted using ImageJ software. Transfection efficiency was calculated as the percentage of EGFP-positive cells relative to total DAPI-positive cells.

### Pharmacological modulation

To probe pathway interactions, cells were treated once at the initiation of odontoblast differentiation with carbenoxolone (CBX; 150 µM; Sigma-Aldrich), a pharmacological modulator reported to affect FOXO activity, or SC79, an AKT activator (2.5 µM; Sigma-Aldrich), as indicated. The concentrations were selected based on previous reports^18,19^ and cell viability results (Supplementary Figure 1), and vehicle-treated cells were used as controls.

### Cell viability assay

HDPCs were seeded in 24-well plates at 2×10⁴ cells per well and incubated at 37^0^C overnight. Different concentrations of carbenoxolone (0 to 200 μM) and SC79 (1 to 5 μM) were administered for 24 h. Cell viability was then assessed using the Quanti-Max™ WST-8 Cell Viability Assay Kit (Biomax, Gyeonggi-Guri, Korea). Briefly, WST-8 was diluted 1:10 in serum-free medium, and 250 μL of diluted WST-8 was added to each well after removal of the culture medium. After incubation at 37^0^C for 1 h, absorbance was measured at 450 nm using a microplate reader (Epoch, BioTek, Winooski, VT, USA).

### Gene expression and Functional assays

Total RNA was extracted from HDPCs using TRIzol reagent^®^ (Invitrogen) according to the manufacturer’s instructions, and reverse transcribed using 3 µg of total RNA and Moloney Murine Leukemia Virus (M-MLV) reverse transcriptase (Promega Corporation, Madison, WI, USA). Quantitative real-time PCR (qRT-PCR) was conducted using SYBR Green PCR^®^ Master Mix (Qiagen N.V., Hilden, Germany), normalized to β-actin (*ACTB*), and analyzed using the 2^-^ method. Specificity was confirmed by melt-curve analysis (technical triplicates), and primer sequences are listed in **Figure 1**. Protein lysates were prepared using CytoBuster Protein Extraction Reagent (Novagen, Madison, WI, USA), and protein expression was analyzed by Western blotting using standard protocols. Primary antibodies (1:1000 dilution) against FOXO3a, phospho-Ser253-FOXO3a, phospho-AKT, AKT, and RUNX2 (Cell Signaling Technology, Danvers, MA, USA), as well as dentin sialophosphoprotein (DSPP), dentin matrix protein (DMP1), and β-actin (Santa Cruz Biotechnology, CA, USA), were used. Alkaline phosphatase (ALP) activity was assessed using an ALP staining kit (Sigma-Aldrich) on day 7 of differentiation, and mineralized matrix formation was evaluated by Alizarin Red S (pH 4.2, Sigma-Aldrich) staining on day 24. For qRT-PCR, ALP, and Alizarin Red S assays, cells from each donor were plated in three independent wells per condition and processed separately. For Western blotting, protein lysates were prepared from one well per donor.

**Figure 1:**
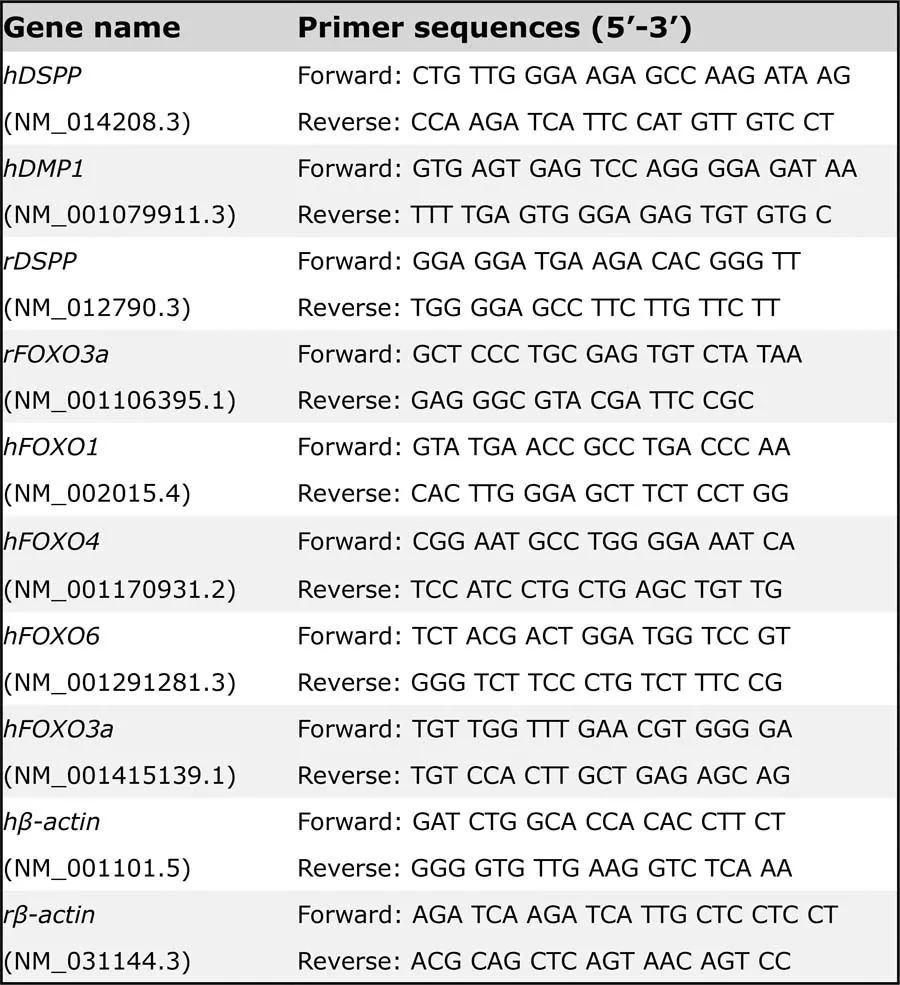
The Primer Sequences used for qRT-PCR analysis.

### Luciferase reporter assay

Promoter activities of RUNX2 and DSPP were evaluated using a luciferase reporter assay system (Promega, Madison, WI, USA). Briefly, approximately 2 × 10⁴ HDPCs were seeded into 24-well plates. Cells were transfected using Lipofectamine LTX reagent (Invitrogen, Carlsbad, CA, USA) with 200 ng of luciferase reporter plasmid containing either the DSPP promoter construct (pGL3-DSPP) or the RUNX2 promoter construct (pGL3-RUNX2), together with 50 ng of the indicated effector plasmid, including FOXO3a or pFLAG-CMV5-Runx2, where appropriate, and 25 ng of a β-galactosidase expression plasmid. The total amount of transfected DNA was kept constant by adding the corresponding empty vector. After 24 h, cells were treated with or without CBX (150 μM) for an additional 24 h. Cells were then harvested, and luciferase activity was measured using a luminometer and normalized to β-galactosidase activity. Cell viability was evaluated under the same transfection conditions, and the results confirmed that the conditions used for the luciferase assay did not significantly affect cell viability (Supplementary Figure 2).

### Animals

Timed-pregnant Sprague Dawley rats were obtained from Damul Science (Daejeon, Korea) to obtain postnatal pups. Dams were maintained under standard laboratory conditions (12:12 h light/dark cycle, 25°C, 50% humidity) with *ad libitum* access to food and water. No specific environmental enrichment was provided. All procedures were approved by the Chonnam National University Animal Care and Use Committee (CNU IACUC-YB-2020-11) and conducted in accordance with institutional and national guidelines. Mandibular molar tissues were harvested from male pups at postnatal days P3, P6, and P9 (n=3 per time point). The pups were derived from at least three independent litters. The pups were clinically assessed as healthy with no overt abnormalities before tissue collection. Body weight was not recorded. Pups were euthanized by CO_2_ inhalation in accordance with the approved protocol, and death was confirmed by cessation of respiration before tissue collection. Pulp tissues were immediately isolated for RNA extraction. The primary outcome for the animal component was FOXO3a mRNA expression in pulp tissues. DSPP expression was assessed as a developmental odontogenic marker. Animals were grouped by predefined postnatal time points (P3, P6, and P9). Each animal was considered an independent experimental unit. Randomization and blinding were not performed because samples were collected based on age-defined developmental stages. qPCR analysis followed pre-specified criteria.

### Statistical Analysis

Student’s t-test was used for comparisons between two groups. One-way analysis of variance (ANOVA) followed by Tukey’s post hoc test was used for comparisons among more than two groups. Biological replicates consisted of three independent donors (n=3). For qRT-PCR, ALP activity, and Alizarin Red S assays, technical triplicates (three wells per donor) were averaged to generate a single value per donor for statistical analysis. For Western blotting, one well per donor was used. Band intensities were quantified by densitometry and normalized to the loading control. Data are presented as mean ± SD. *P*-values < 0.05 were considered statistically significant. For animal qRT-PCR data, the unit of analysis was a single animal. Given the small sample size, non-parametric tests were used. Differences among time points were evaluated using the Kruskal–Wallis test followed by Dunn’s multiple comparisons test. For comparisons between two groups, the Mann–Whitney U test was applied. Statistical analyses were performed using GraphPad Prism (version 5.0, GraphPad Software, San Diego, CA, USA).

## Results

### FOXO3a Expression Increases During Odontoblast Differentiation of HDPCs

To investigate the expression of FOXO family members in HDPCs, qRT-PCR analysis was performed. Among FOXO family transcription factors, *FOXO3a* exhibited the highest expression in HDPCs and was selected for further analysis (**Figure 2A**). To assess its *in vivo* expression pattern, developing postnatal rat teeth were analyzed at defined stages. The second molar at postnatal days 3, 6, and 9 corresponds to the late cap, bell, and crown stages, respectively, whereas the third molar at postnatal day 3 represents the early cap stage.^20^ *Foxo3a* and *Dspp* mRNA levels were quantified by qRT-PCR in pulp tissues from the second molar at P3, P6, and P9 and from the third molar at P3 (n=3 animals per group). No animals were excluded from the analysis, and no adverse events were observed. qRT-PCR analysis revealed that *Dspp* expression was progressively increased in the second molar pulp, whereas *Foxo3a* expression was peaked at postnatal day 6 (**Figure 2B**). During *in vitro* odontoblast differentiation of HDPCs, *FOXO3a* mRNA levels gradually increased relative to day 0 (**Figure 2C**). Western blot analysis showed an increase in FOXO3a protein expression at day 3 (**Figure 2D).**

**Figure 2:**
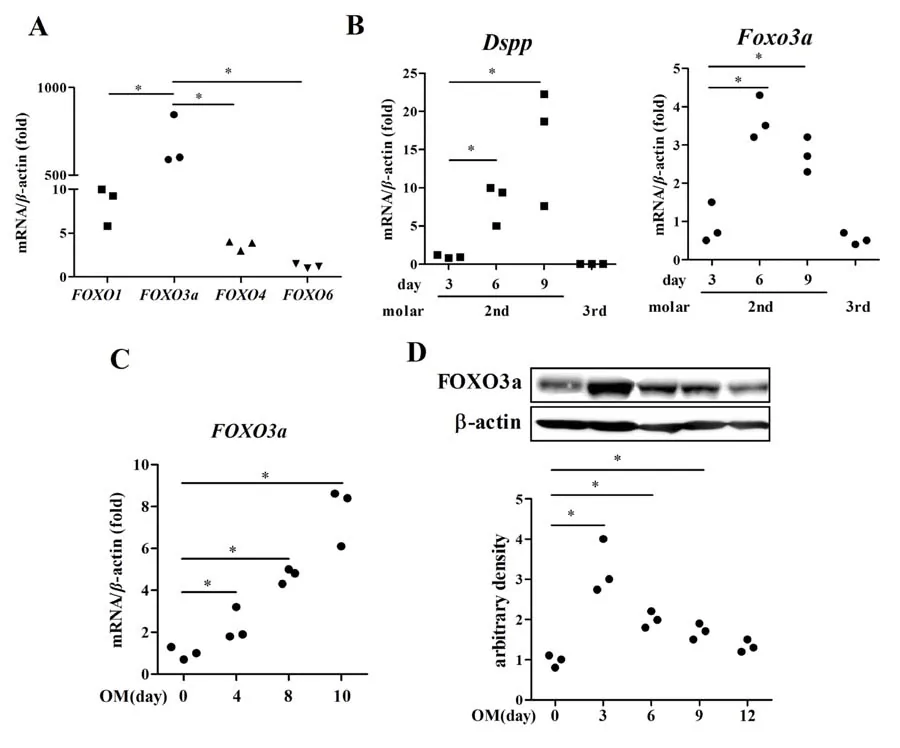
FOXO3a expression during odontogenic differentiation. (A) mRNA expression levels of FOXO family members in HDPCs were quantified by qRT-PCR. (B) *Dspp* and *Foxo3a* mRNA expression levels were analyzed by qRT-PCR in postnatal rat second molars at defined developmental stages, with the third molars used for comparison. (C) *FOXO3a* mRNA expression was measured in HDPCs cultured in odontogenic differentiation medium (OM) for 4, 8, and 10 days. (D) FOXO3a protein expression during odontogenic differentiation was assessed by Western blotting at the indicated time points. Data are presented as mean ± standard deviation (SD) from three independent donors or three animals per time point.

### FOXO3a Suppression Inhibits Odontoblast Differentiation

The functional role of FOXO3a was assessed using loss-of-function approaches. *FOXO3a* mRNA expression was significantly reduced by approximately 43.8% following *FOXO3a* siRNA transfection compared with the control group, confirming *FOXO3a* knockdown (**Figure 3A**). After 7 days of differentiation, *DSPP* and *DMP1* mRNA expression levels were significantly reduced in *FOXO3a*-silenced HDPCs compared with control cells (**Figure 3B**). At later differentiation time points, DSPP protein expression was significantly decreased on days 9 and 12 of differentiation (**Figure 3C**). RUNX2 protein expression was also significantly decreased on day 5 of differentiation following *FOXO3a* knockdown (**Figure 3D**). *FOXO3a* knockdown also reduced ALP activity and mineralized matrix formation, as demonstrated by ALP and Alizarin Red S staining (**Figure 3E**). Similar inhibitory effects were observed following treatment with carbenoxolone (CBX), a reported modulator of FOXO activity (**Figure 3F**).

**Figure 3:**
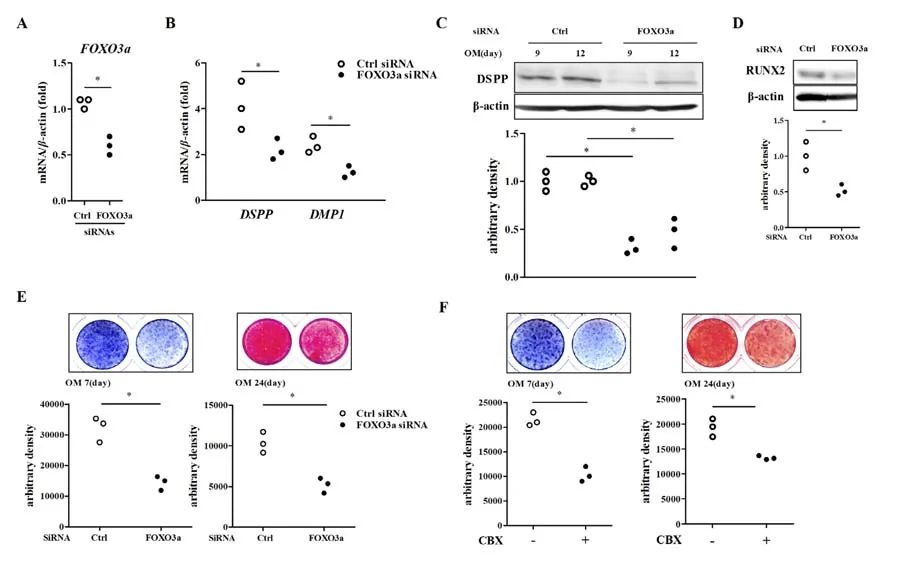
Effect of FOXO3a knockdown or inhibition on odontoblast differentiation. (A) HDPCs were transfected with control or FOXO3a-specific siRNA, and *FOXO3a* mRNA expression was quantified by qRT-PCR. (B) After 7 days of odontoblast differentiation, *DSPP* and *DMP1* mRNA expression levels were measured by qRT-PCR. (C, D) Protein expression levels of DSPP and RUNX2 were analyzed by Western blotting. (E) ALP activity and matrix mineralization were assessed by ALP and Alizarin Red S staining following FOXO3a knockdown. (F) Effects of carbenoxolone (CBX) on odontoblast differentiation were evaluated by ALP and Alizarin Red S staining. Data are presented as mean ± SD from three independent donors.

### FOXO3a Overexpression Promotes Odontoblast Differentiation

To complement the loss-of-function analysis, HDPCs were transfected with a *FOXO3a* overexpression plasmid. qRT-PCR analysis confirmed a significant increase in *FOXO3a* mRNA expression compared with the control group (**Figure 4A**). Plasmid transfection efficiency was assessed using an EGFP-expressing control plasmid under the same transfection conditions and is shown in Supplementary Figure 3. Under odontogenic differentiation conditions, *FOXO3a* overexpression significantly increased *DSPP* and *DMP1* expression at both mRNA and protein levels compared with controls (**Figures 4A** and **4B**). Furthermore, *FOXO3a* overexpression increased RUNX2 protein levels (Figure 4C). Consistent with the loss-of-function results, *FOXO3a* overexpression enhanced ALP activity and mineralized matrix formation, as demonstrated by Alizarin Red S staining (**Figure 4D**).

**Figure 4:**
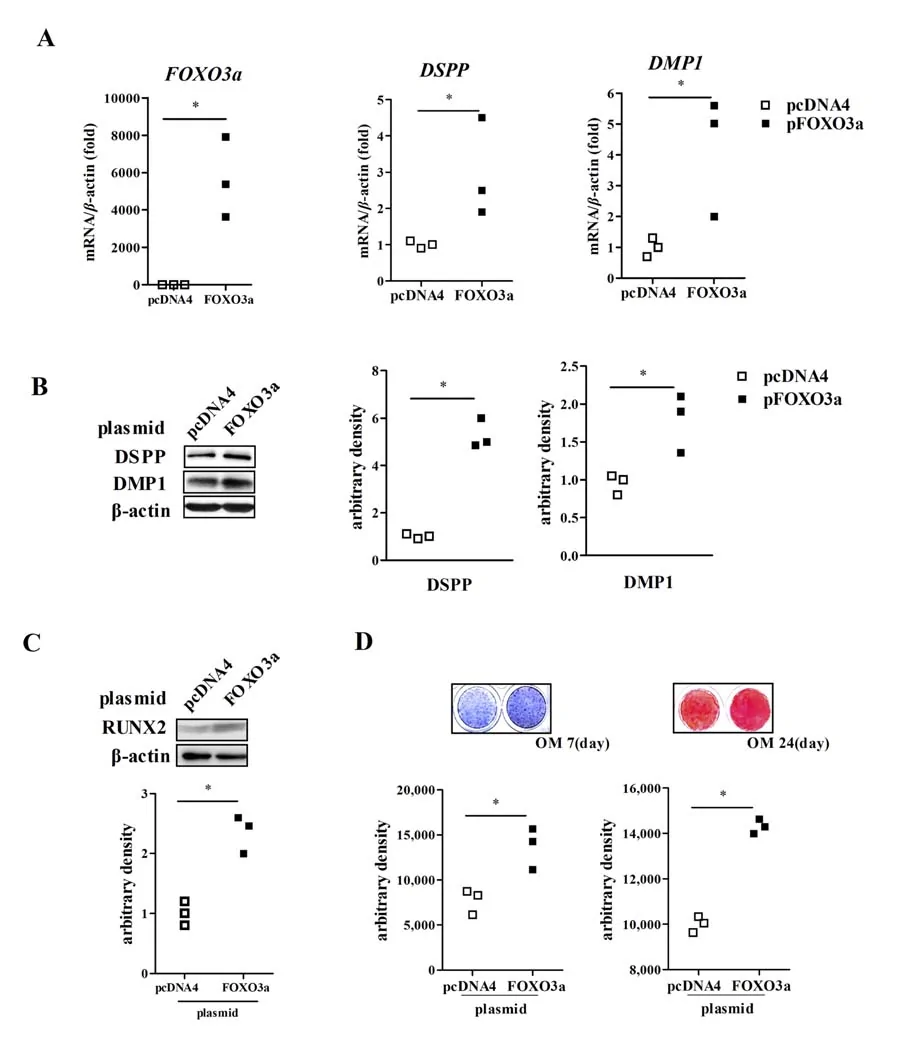
Effect of FOXO3a overexpression on odontoblast differentiation. HDPCs were transfected with a FOXO3a expression plasmid and cultured after 24 h in odontogenic differentiation medium (OM). (A) *FOXO3a, DSPP,* and *DMP1* mRNA expression levels were analyzed by qRT-PCR. (B, C) Protein expression levels of DSPP, DMP1, and RUNX2 were evaluated by Western blotting. (D) ALP activity and matrix mineralization were assessed by ALP and Alizarin Red S staining. Data are presented as mean ± SD from three independent donors.

### FOXO3a Is Associated with RUNX2-Dependent Promoter Activity and AKT Signaling During Odontoblast Differentiation

To investigate the mechanism underlying FOXO3a-mediated regulation of dentin matrix gene expression, luciferase reporter assays were performed. RUNX2 overexpression significantly increased *DSPP* promoter activity compared with the control group. Co-expression of FOXO3a with RUNX2 further enhanced *DSPP* promoter activity, whereas FOXO3a overexpression alone had only minimal effects (**Figure 5A**). This enhancement was attenuated by CBX treatment (**Figure 5B**). *FOXO3a* overexpression also increased *RUNX2* promoter activity, and this effect was reduced by CBX (**Figure 5C**). To examine phosphorylation-dependent regulation during odontoblast differentiation, Western blotting analysis was performed. FOXO3a phosphorylation at serine 253, a modification associated with AKT-mediated regulation,^21^ showed dynamic changes over the differentiation time course. The pFOXO3a/FOXO3a ratio initially decreased at the early stage of differentiation compared with day 0 and subsequently increased. In contrast, the pAKT/AKT ratio showed a gradual increase throughout differentiation from day 0 onward, suggesting a differential temporal relationship between FOXO3a phosphorylation and AKT activation during odontoblast differentiation (**Figure 5D**). In *FOXO3a*-silenced HDPCs, the pAKT/AKT ratio was reduced relative to controls. However, a gradual increase was observed over differentiation days 3, 6, 9, and 12 (**Figure 5E**). Treatment with SC79 significantly increased the mRNA expression of *DSPP* and *DMP1* (**Figure 5F**). DSPP protein expression was also increased by SC79 (**Figure 5G**). In *FOXO3a*-silenced HDPCs, SC79 partially restored *DSPP* and *DMP1* mRNA expression after 7 days of differentiation (**Figure 5H**). SC79 also increased DSPP protein expression under these conditions (**Figure 5I**). These findings suggest that AKT activation partially restores odontogenic marker expression in *FOXO3a*-silenced HDPCs.

**Figure 5:**
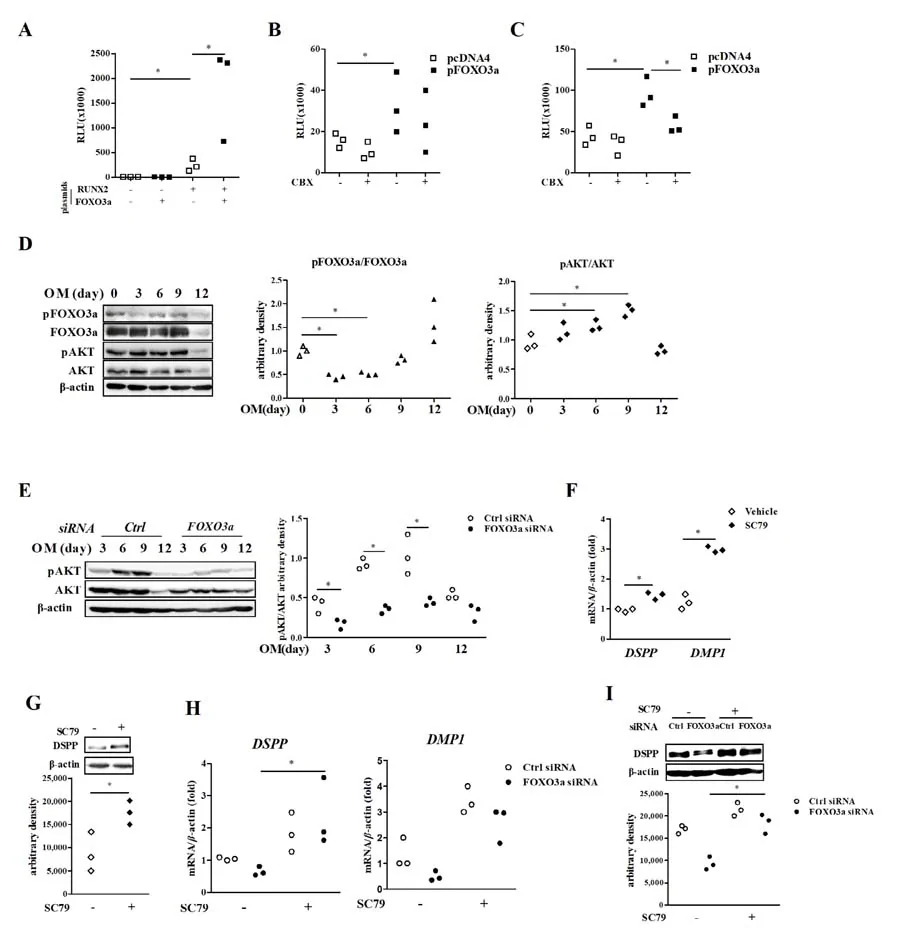
FOXO3a is associated with RUNX2-dependent promoter activity and AKT signaling during odontoblast differentiation. (A) *DSPP* promoter activity was assessed using luciferase reporter assays in HDPCs transfected with RUNX2 and/or FOXO3a expression plasmids. (B) HDPCs were co-transfected with the DSPP promoter construct, RUNX2, and FOXO3a expression plasmids for 18 h, followed by treatment with carbenoxolone (CBX) for an additional 24 h. Luciferase activity was measured and normalized to β-galactosidase activity. (C) *RUNX2* promoter activity was evaluated following transfection of cells with FOXO3a expression plasmids. (D) Protein levels of phospho-FOXO3a (Ser253) and phospho-AKT during odontoblast differentiation were analyzed by Western blotting. (E) HDPCs transfected with control or FOXO3a siRNA were cultured under odontogenic differentiation conditions, and protein levels of phospho-AKT and total AKT were analyzed by Western blotting. (F) HDPCs were treated with SC79, an AKT activator, during odontoblast differentiation, and *DSPP* and *DMP1* mRNA expression was quantified by qRT-PCR. (G) DSPP protein expression following SC79 treatment was analyzed by Western blotting. (H, I) Combined effects of FOXO3a knockdown and SC79 treatment on *DSPP* and *DMP1* expression were evaluated by qRT-PCR and Western blotting, respectively. Data are presented as mean ± SD from three independent donors.

## Discussion

FOXO3a has been implicated in regulating cellular differentiation and stress-responsive transcriptional programs across multiple tissues; however, its role in odontoblast differentiation has remained largely unexplored.^22,23^ Given the partially overlapping yet distinct molecular frameworks governing osteogenesis and odontogenesis, direct extrapolation of FOXO3a function between these lineages should be undertaken with caution. Although FOXO3a has been implicated in osteoblast differentiation and bone homeostasis,^7,8,24^ whether it participates in dentin matrix gene regulation within the odontoblastic lineage has not been directly examined. In this context, the present study investigated the role of FOXO3a in odontoblast differentiation and its functional relationship with key odontogenic regulators.

In this study, FOXO3a expression was elevated during the early phases of odontoblast differentiation in both postnatal rat molars and differentiating HDPCs. Although *FOXO3a* mRNA levels increased progressively during differentiation, FOXO3a protein expression peaked during the early stages, suggesting that FOXO3a may be regulated beyond the transcriptional level. While these findings do not establish a causal role for FOXO3a at specific differentiation stages, they support its functional relevance during the initiation of odontoblast differentiation. Our loss- and gain-of-function models suggest that, rather than functioning merely as a passive downstream survival factor, FOXO3a may serve as a regulatory component of the odontogenic transcriptional program. The significant reduction in dentin matrix gene expression and mineralized matrix formation following *FOXO3a* knockdown, together with the reciprocal effects observed after *FOXO3a* overexpression, suggests that FOXO3a contributes to maintaining an odontogenic gene expression program required for functional odontoblast differentiation. This pro-odontoblastic role supports the concept that FOXO3a may exert lineage-dependent effects in mineralized tissues. Interestingly, this finding is consistent with the pro-osteogenic, SIRT1-mediated mechanisms previously observed in bone homeostasis, where FOXO3a activation supports osteoblast survival and function.^7^ However, our findings differ from observations in some osteoblastic models in which FOXO3a has been reported to restrain osteogenesis or in which FOXO inactivation favored bone formation.^8^ This phenotypic discrepancy suggests that FOXO3a functions in a tissue-specific regulatory context. Unlike the continuously remodeling bone microenvironment, the dental pulp represents a specialized niche involved in dentin formation and repair. Thus, FOXO3a may act as a context-dependent regulator whose functional outcome is shaped by the cellular niche and interacting lineage-specific factors.

Mechanistically, FOXO3a increased *RUNX2* promoter activity and cooperated with RUNX2 to regulate *DSPP* promoter activity. *FOXO3a* alone was insufficient to activate the *DSPP* promoter, suggesting that FOXO3a potentiates RUNX2-mediated transcription rather than acting as an independent driver of *DSPP* promoter activity. This mode of regulation is particularly relevant during odontoblast differentiation, where RUNX2 functions as an early-stage regulator of dentin matrix gene expression. Although ChIP assays were not performed and direct binding of FOXO3a to the *RUNX2* or *DSPP* promoter was not demonstrated, these findings support a model in which FOXO3a modulates RUNX2-mediated transcription, consistent with reported roles of FOXO family members as transcriptional modulators.^7^

Our downstream signaling data provide insight into the temporal regulation of FOXO3a during HDPC differentiation. FOXO3a activity is regulated by AKT-mediated phosphorylation, which promotes cytoplasmic retention and functional inactivation.^25,26^ While continuous AKT-mediated suppression of FOXO3a is often considered a negative regulatory mechanism in certain osteoblast models, our data captured a more dynamic, stage-specific phosphorylation pattern during HDPC commitment. Notably, the transient reduction in FOXO3a Ser253 phosphorylation during the early stage of differentiation suggests a temporal window that may favor FOXO3a nuclear activity and contribute to the early odontogenic transcriptional program. In addition, the finding that AKT activation via SC79 could partially rescue the phenotype of *FOXO3a*-silenced cells suggests that FOXO3a may act within a broader pro-odontogenic signaling network, rather than serving as the only mediator of this process**.** Given the broad downstream effects of AKT, these findings are more consistent with the involvement of parallel or FOXO3a-independent pro-odontogenic pathways than with functional substitution of FOXO3a by AKT activation. Thus, although the present data suggest a functional interplay between FOXO3a status and AKT signaling during differentiation, they do not clarify whether FOXO3a acts upstream of AKT in a feedback loop. Alternatively, FOXO3a and AKT may function through parallel pro-odontogenic pathways.

SIRT1- and SIRT3-related pathways have been implicated in bone homeostasis, suggesting that FOXO3a coordination with RUNX2 in HDPCs may also involve sirtuin-mediated or redox-sensitive mechanisms. Sirtuins are known to deacetylate FOXO3a,^27-29^ thereby altering its binding affinity and shifting its transcriptional targets toward genes involved in cell survival and differentiation. Indeed, all sirtuin isoforms, including the mitochondrial deacetylase SIRT3, have been shown to be actively and continuously expressed during the odontoblastic differentiation of HDPCs.^30^ Notably, recent evidence has supported the functional significance of the SIRT3/FOXO3 axis in dental pulp stem cells, demonstrating that SIRT3 upregulation enhances the FOXO3 pathway through deacetylation-dependent mechanisms, thereby promoting odontogenic differentiation and dentin repair.^31^ During the energy-demanding process of biomineralization, HDPCs experience dynamic shifts in reactive oxygen species (ROS) levels. FOXO3a, potentially influenced by SIRT1/3-dependent modification, may function as a transcriptional modulator that links RUNX2-associated dentin matrix gene expression with stress-adaptive signaling. Consideration of these sirtuin-mediated frameworks may provide a broader interpretation of how FOXO3a connects the metabolic state, redox balance, and lineage-specific transcription during biomineralization.

Collectively, these findings provide functional evidence that FOXO3a contributes to odontoblast differentiation in coordination with RUNX2-associated transcriptional activity and dynamic regulation by AKT signaling. In contrast to FOXO1, which has been reported to function as a transcriptional regulator in ameloblasts downstream of TGF-β signaling and to modulate amelogenesis-related gene expression as a cofactor, FOXO3a appears to be a distinct regulatory component of the pulp–dentin complex.^32^ Thus, while FOXO family members may share a general capacity to modulate lineage-specific transcriptional programs, the present findings suggest that FOXO3a acts in a cellular and developmental context distinct from the FOXO1-mediated regulation of enamel formation. By framing these observations in light of known FOXO roles in amelogenesis and bone biology, this study extends the interpretation of FOXO3a beyond a simple differentiation-associated marker and supports its role as a context-dependent regulator of odontoblast differentiation within the pulp–dentin complex. Although the present findings support a transcriptional role for FOXO3a during odontoblast differentiation, it remains unclear whether FOXO3a directly binds to the regulatory regions of odontogenic genes such as *RUNX2* and *DSPP*, or exerts its effects indirectly through interaction with other transcriptional regulators. Future studies using ChIP-based and genome-wide approaches will be required to determine whether FOXO3a directly regulates odontogenic gene loci.

This study has several limitations. In the absence of FOXO3a-specific inhibitors, a pharmacological agent reported to affect FOXO activity was used. Direct genomic targets of FOXO3a were not identified. Although the HDPCs used in this study were previously characterized by flow cytometry, future lineage-specific flow cytometric analyses will be needed to define subpopulation-specific responses during FOXO3a-mediated odontogenic differentiation. *In vivo* analyses were restricted to expression profiling, and animal use was minimized in accordance with the 3Rs principle (Replacement, Reduction, and Refinement). Caution should be exercised when translating these findings to human dentin–pulp biology, as species- and developmental stage–specific differences may exist. These limitations should be addressed in future studies to further define the role of FOXO3a in dentin–pulp regeneration.

## Conclusion

FOXO3a promotes odontoblast differentiation of HDPCs and regulates *DSPP* expression in coordination with RUNX2-associated transcriptional activity. These findings identify FOXO3a as a potential regulatory component of dentin matrix gene expression relevant to dentin–pulp regeneration.

## Supplementary Figures

Supplementary Figure 1Effects of carbenoxolone and SC79 on HDPC viability.
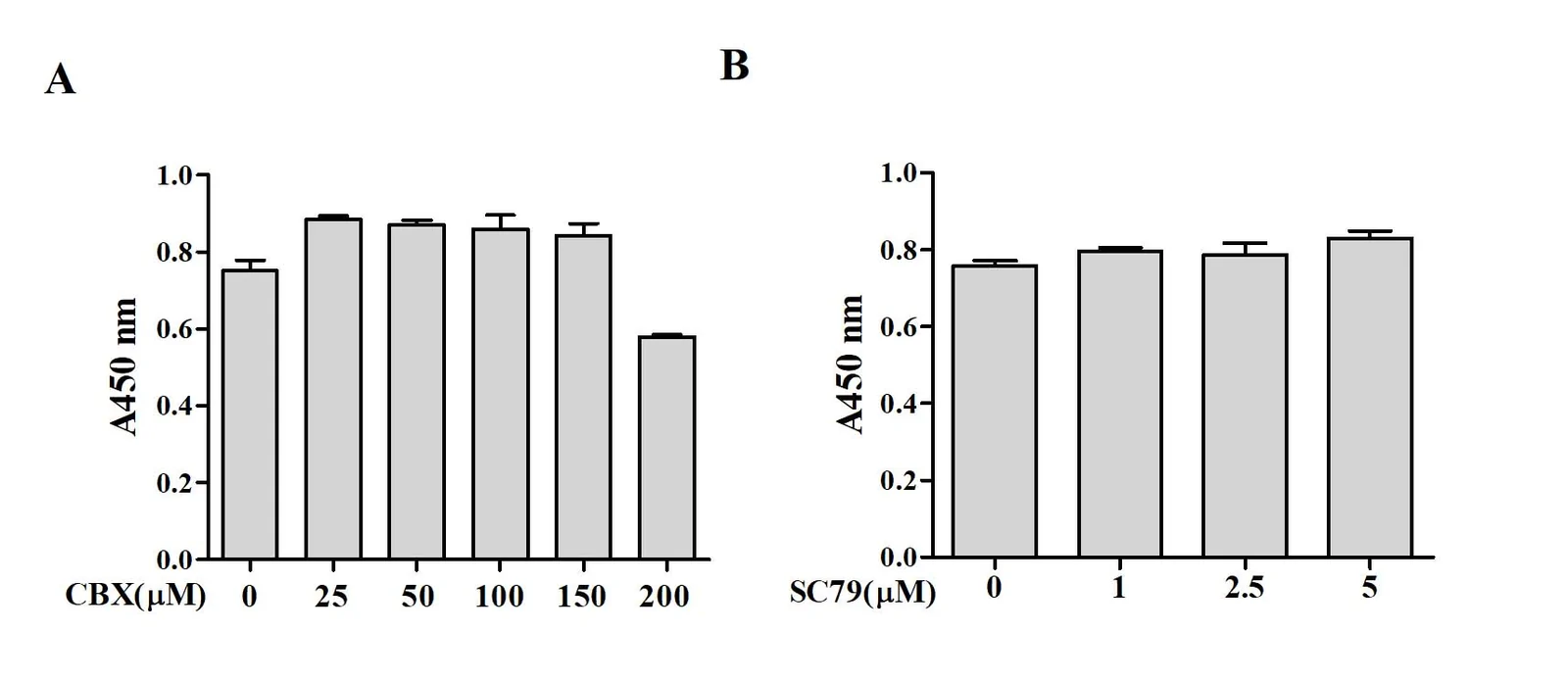
In Replication Data for: FOXO3a promotes odontoblast differentiation in human dental pulp cells in coordination With RUNX2 (version 1.0), by Kim, Min-Seok; Son, Su-Kyung; Moon, Jung-Sun; Jung, Na-Ri; Kang, Jee-Hae; Lee, Bin-Na; Kim, Sun-Hun

Supplementary Figure 2Cell viability under luciferase reporter assay transfection conditions.
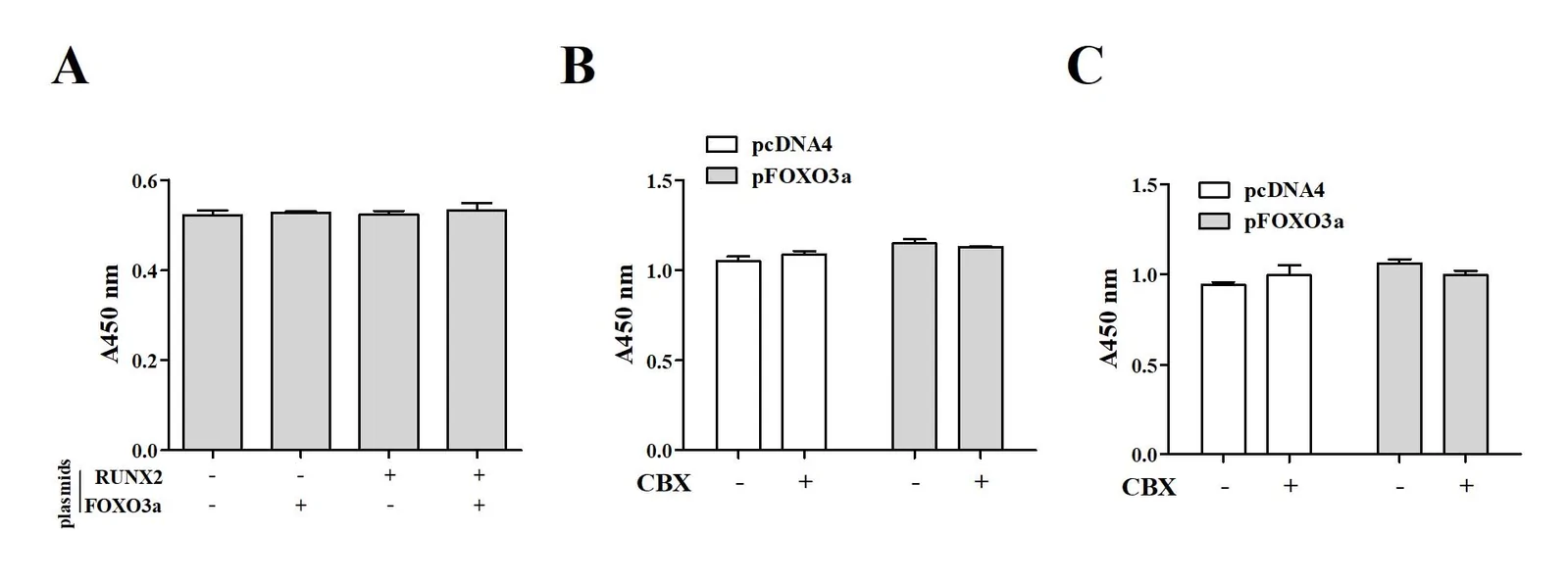
In Replication Data for: FOXO3a promotes odontoblast differentiation in human dental pulp cells in coordination With RUNX2 (version 1.0), by Kim, Min-Seok; Son, Su-Kyung; Moon, Jung-Sun; Jung, Na-Ri; Kang, Jee-Hae; Lee, Bin-Na; Kim, Sun-Hun

Supplementary Figure 3Transfection efficiency of the overexpression vector in HDPC.
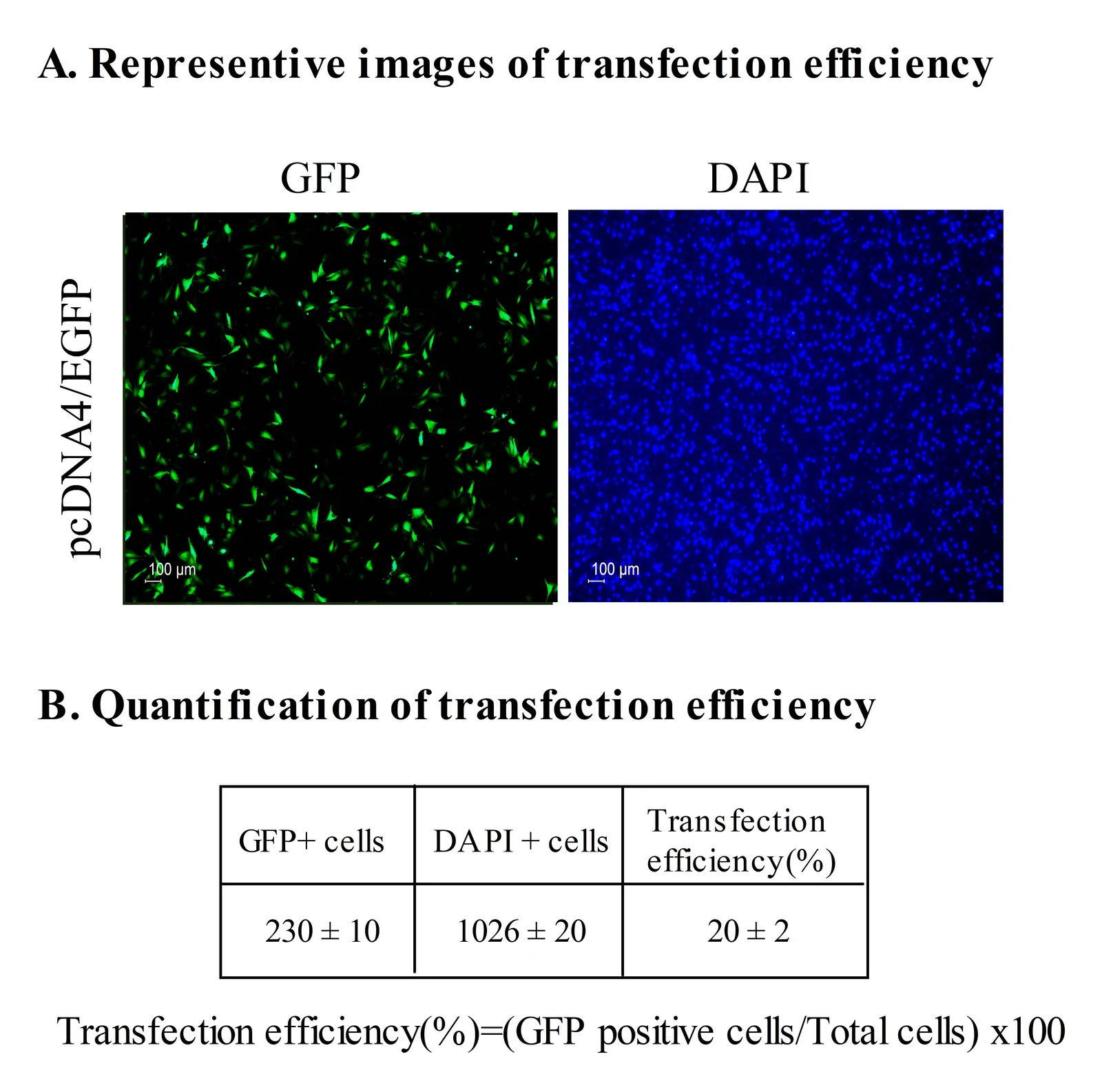
In Replication Data for: FOXO3a promotes odontoblast differentiation in human dental pulp cells in coordination With RUNX2 (version 1.0), by Kim, Min-Seok; Son, Su-Kyung; Moon, Jung-Sun; Jung, Na-Ri; Kang, Jee-Hae; Lee, Bin-Na; Kim, Sun-Hun
